# Medial pivot prosthesis has a better functional score and lower complication rate than posterior-stabilized prosthesis: a systematic review and meta-analysis

**DOI:** 10.1186/s13018-022-03285-0

**Published:** 2022-08-19

**Authors:** Weipeng Shi, Yaping Jiang, Yingzhen Wang, Xuan Zhao, Tengbo Yu, Tao Li

**Affiliations:** 1 Department of Orthopaedic Surgery, The Affiliated Hospital of Qingdao University, No. 59, Haier Road, Qingdao, 266000 China; 2 Medical Department of Qingdao University, Qingdao, 266071 Shandong China; 3 Department of Oral Implantology, The Affiliated Hospital of Qingdao University, Qingdao, 266003 China; 4 Department of Rheumatism and Immunology, The Affiliated Hospital of Jining Medical University, Jining, 272007 China

**Keywords:** Medial pivot, Posterior-stabilized, Knee osteoarthritis, Total knee arthroplasty, Meta-analysis

## Abstract

**Purpose:**

We aimed to compare the postoperative clinical efficacy and safety of medial pivot (MP) prosthesis and posterior-stabilized (PS) prosthesis in the treatment of knee osteoarthritis (KOA).

**Methods:**

All studies involving MP and PS prosthesis in PubMed, EMBASE, Cochrane Library, and Web of Science were searched since the establishment of the database. The included outcomes were knee range of motion (ROM), functional score, radiographic results, complication rate, and revision rate. Studies were independently evaluated by the Newcastle–Ottawa Scale for case–control studies and the assessment tool of the Cochrane Collaboration for randomized controlled trials. *I*^2^ was used to test the heterogeneity, and fixed- or random-effects models were selected for meta-analysis according to the heterogeneity results.

**Results:**

A total of 19 studies, consisting of 3592 patients and 3783 knees (MP: 1811 knees, PS: 1972 knees), were included in the meta-analysis. The WOMAC (MD = − 1.11, 95% CI − 1.98 to − 0.23; *P* = 0.01) and HSS (MD = − 4.32, 95% CI − 8.30 to − 0.34; *P* = 0.03) in the MP group were significantly lower compared with the PS group, and the complication rate (OR 0.53, 95% CI 0.33–0.87; *P* = 0.01) was also lower compared with the PS group. There was no significant difference in ROM, radiographic results, and revision rate between the two groups (*P* > 0.5).

**Conclusions:**

The existing literature provided evidence to support better clinical effect and lower complication rate of MP prosthesis compared to PS prosthesis. These results provide a reference for clinicians when choosing a suitable prosthesis.

## Introduction

As an effective treatment for end-stage knee osteoarthritis (KOA), total knee arthroplasty (TKA) has been increasingly performed year by year. One study has predicted that the demand for primary TKA in the USA will increase by 673% to 3.48 million by 2030 [1]. At present, the overall effect of TKA is satisfactory, while 10–15% of patients are still disappointed [2], especially young people with greater exercise demand [3]. One important reason for dissatisfaction is the change in knee kinematics, as well as pain, which is an important risk factor related to patient satisfaction [2, 4]. For doctors, selecting an optimal prosthesis is also an important issue that will influence patient outcomes. Posterior-stabilized (PS) prosthesis relies on a cam–column to achieve knee motion and stability instead of the posterior cruciate ligament, and it improves knee flexion enhancing the roll-back motion of the femur [5, 6]. However, during mid-flexion of the PS prosthesis, the femur will slide forward and may present “paradoxical anterior movement” [7], and only when the cam and column collide, the ideal “roll-back” will be achieved, which is a potential cause of knee mid-flexion instability. In addition, the design of a multi-curvature radius may lead to unstable soft tissue tension and affect the stability of the knee. Moreover, anterior knee pain (AKP) and patellar clunk or crepitus (PCC) are also common complications of PS prosthesis [8].

The medial side of the medial pivot (MP) prosthesis is designed as a "ball-socket," which constrains the movement of the medial compartment, while the lateral compartment can move forward and backward relatively freely. The MP prosthesis has a single curvature radius that enhances strength of the quadriceps femoris, ensures constant tension of the lateral collateral ligament throughout flexion [9], and raised anterior and posterior lips of polyethylene insert for enhancing joint stability. Polyethylene wear is one important reason for revision of TKA [10], whereas the MP prosthesis reduces contact stress, polyethylene wear, and improves prosthesis survival rate by maximizing the contact area between polyethylene insert and femoral prosthesis. Besides, the MP prosthesis does not need an intercondylar box to accommodate columns, and such a design is beneficial to reducing bone loss and the incidence of AKP and PCC [8, 11].

Up to now, there are still disputes about the postoperative effects of the two prostheses, and only a few meta-analyses have analyzed these problems. No significant difference in the results of these previous analyses could be due to the fact that fewer studies or outcomes were included [12–15]. In particular, the complications have not been fully analyzed in these analyses. As we all known, many factors in orthopedic surgery could lead to postoperative complications. Serious complications, such as periprosthetic joint infection (PJI), will bring catastrophic consequences for patients, so reducing complication and revision rates is important. Therefore, we aimed to make a further comprehensive evaluation of MP prosthesis and PS prosthesis to provide a reference for optimizing the selection of prostheses.

## Materials and methods

The study was planned according to the preferred reporting items for systematic reviews and meta-analyses (PRISMA) statement [16].

### Search strategy

We searched four databases (PubMed, Embase, Cochrane Library, and Web of Science) for the published literature from the establishment of the database to September 2021. Search terms included media pivot, media rotating, media ball and socket, media-stabilized, MP, posterior-stabilized, PS, total knee arthroplasty, total knee replacement, TKA, and TKR. Moreover, we also searched the references of related literature to reduce the loss of information. When there were multiple studies in the same group, we included data from the most recent one. There were no language restrictions.

### Inclusion and exclusion criteria

All studies included in the meta-analysis met the following criteria: (1) patients receiving primary TKA; (2) clinical studies comparing the efficacy of MP-TKA and PS-TKA, and (3) outcomes included at least one of the following outcomes: range of motion (ROM), knee function score, radiographic results, and complications.

Exclusion criteria were set up as follows: (1) review, case report, comment, or letter; (2) in vitro study; (3) duplicate literature; (4) no MP-TKA and PS-TKA comparison or no control group; and (5) inability to acquire valid data.

### Data extraction

The title, abstract, and full text of the included studies were read and evaluated by two researchers, and then the data were extracted according to the data table formulated earlier:

1. *Literature information* first author's last name, year of publication, and type of study;
2. *Baseline data* sample size, age, sex ratio, body mass index (BMI), and prosthesis type;
3. *Follow-up outcomes* last follow-up time, ROM, functional score including knee society score (KSS), the Western Ontario and McMaster Universities (WOMAC) osteoarthritis, Oxford Knee Score (OKS), Hospital for Special Surgery scoring system (HSS) score and forgotten joint score (FJS), radiographic results (α. the angle between the tangent line of the medial and lateral condyles of the femoral component on the coronal plane and the anatomical axis of the femur; β. the angle between the lower edge of the tibial component and the anatomical axis of the tibia on the coronal plane; γ. the angle between the perpendicular line of the femoral condyle tangent and the femoral anatomical axis in the sagittal plane; δ. the angle between the lower edge of the tibial component and the anatomical axis of the tibia in the sagittal plane) [17], complications (local complications of the knee: AKP, PCC, superficial or deep infection, numbness around the incision, recurrent effusion, hematoma and wound dehiscence; prosthesis-related complications: PJI, periprosthetic fracture, aseptic loosening, and knee instability; systemic complications: deep venous thrombosis, pulmonary embolism, acute myocardial infarction), and revision.

If the data could not be extracted, we contacted the authors by email to obtain the original data. When the evaluations of the both researchers were inconsistent, the final decision was made through discussion and consultation with the third researcher.

### Assessment of risk of bias (ROS)

The ROS was assessed by two researchers. The quality of randomized controlled trials (RCTs) was assessed by the Cochrane risk bias assessment tool. Green, yellow, and red represented a low, unknown, and high risk, respectively. The case–control studies were assessed by Newcastle Ottawa Scale (NOS), with 0–3 as low quality, 4–6 as medium quality, and 7–9 as high quality.

### Statistical analysis

The Review Manager Version 5.3 (Cochrane Collaboration, Copenhagen, Denmark) was used for statistical analysis. The heterogeneity of the literature was evaluated by the Cochrane Q test and *I*^2^. *P* < 0.1 or *I*^2^ > 50% considered significant heterogeneity, and if there was still heterogeneity after sensitivity analysis, the random-effects model was used for meta-analysis. *P* > 0.1 and *I*^2^ < 50% indicated that there was no heterogeneity, and the study was assessed by the fixed-effects model. Continuous variables were described by mean difference (MD) and 95% confidence interval (CI), and binary data were described by odds ratio (OR) and 95% CI. A funnel plot was used to analyze whether there was a publication bias in the included studies, and all results were presented by forest plots.

## Results

### Search results

**Fig. 1:**
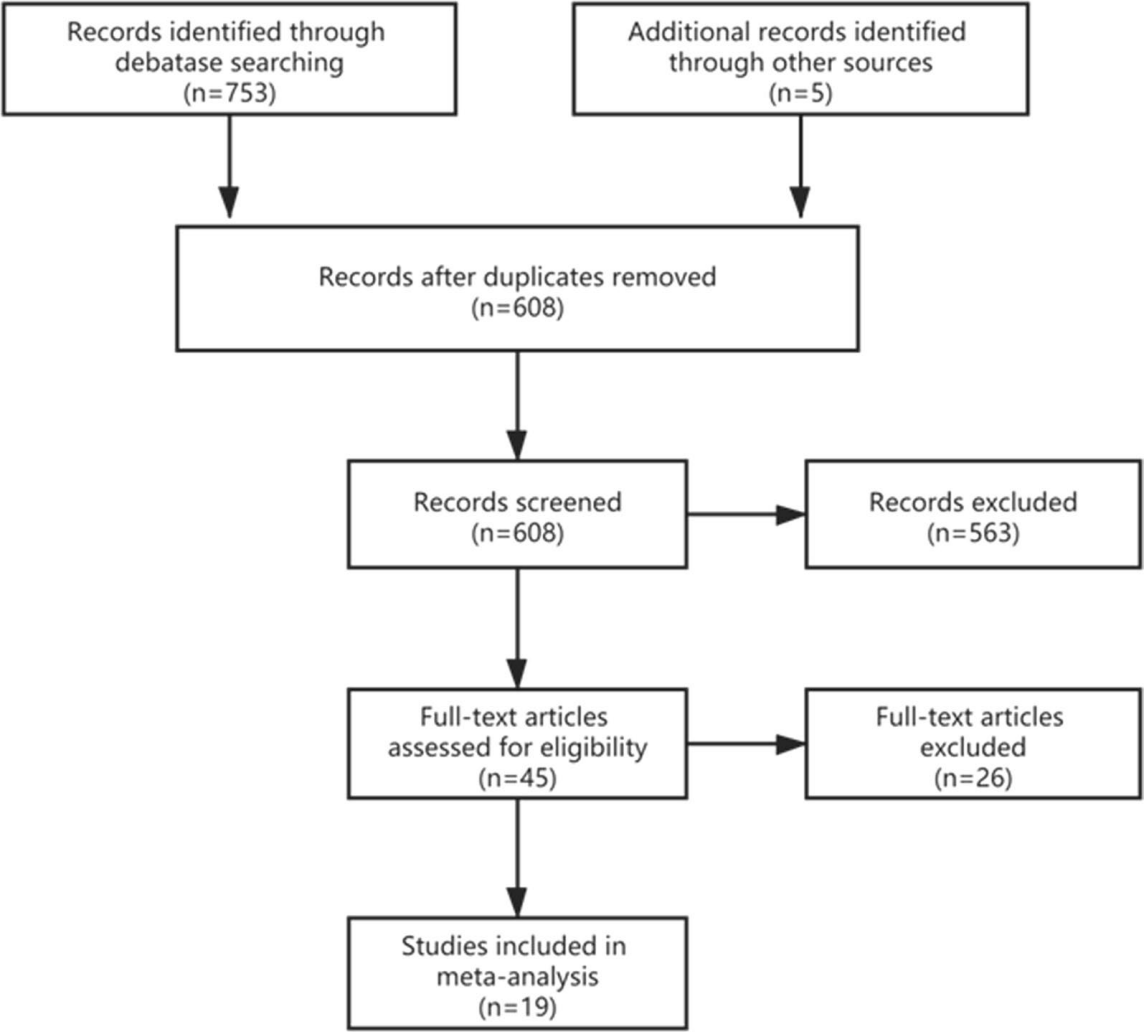
Flow chart of meta-analysis

According to the search strategy, 753 studies were identified from the databases, and five references met the inclusion criteria, and a total of 758 studies were included in the preliminary review. In total, 150 duplicate studies were excluded firstly. After the two researchers read the title and abstract, they further ruled out 563 irrelevant studies. Finally, the researchers reviewed the full text of the remaining 45 studies and determined that 19 studies could be included in the final meta-analysis [4, 5, 8, 11, 18–31]. Figure 1 shows the flow chart.

### Baseline characteristics and ROS of the included studies

**Fig. 2:**
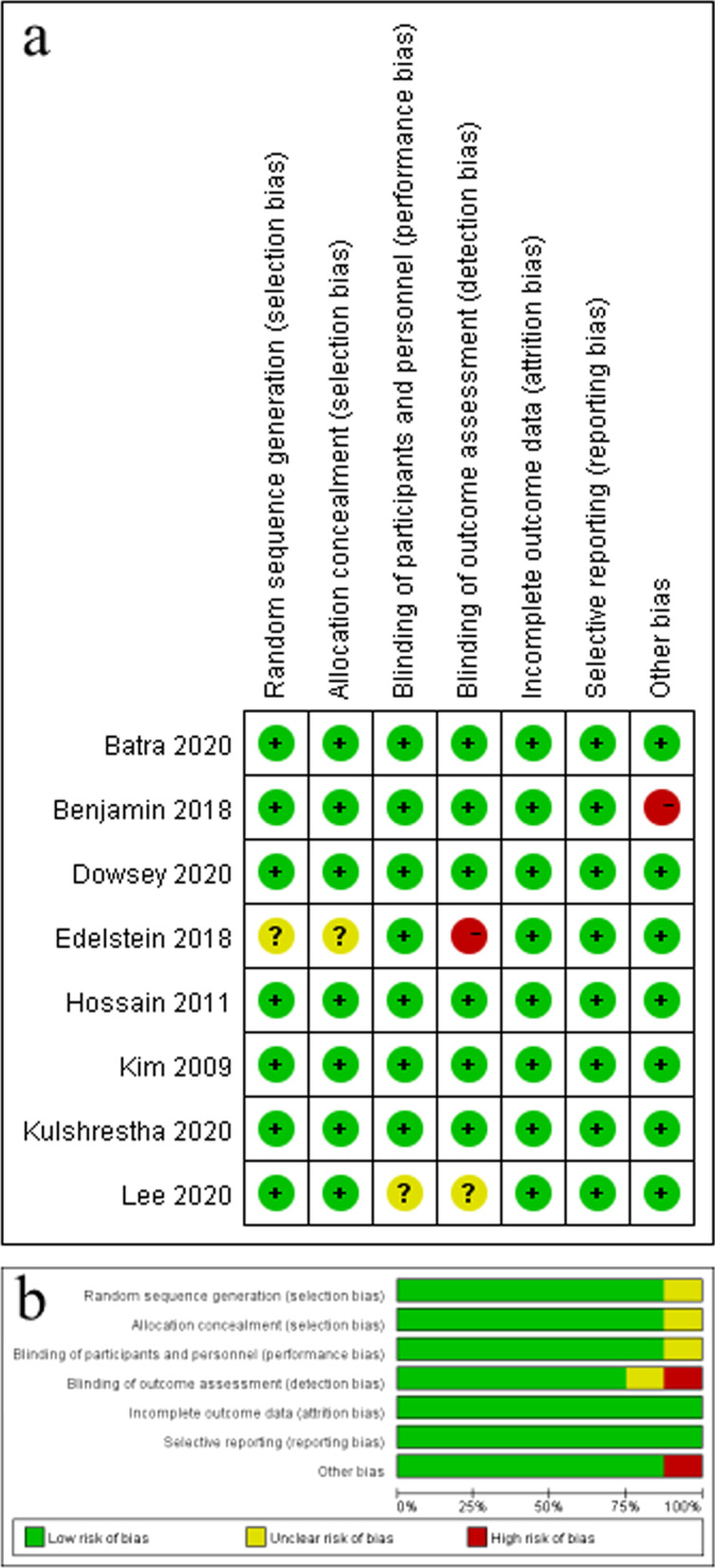
Risk of bias summary. **a** RCT evaluation chart of bias risk analysis items, " + " represents a low risk, "-" represents a high risk, and "?" represents an unknown risk; **b** RCT risk of bias graph, green: low risk, yellow: unknown risk, and red: high risk

A total of 19 studies consisting of 3592 patients and 3783 knees (MP: 1811 knees, PS: 1972 knees) were included in this meta-analysis, including eight RCTs [18, 19, 21, 22, 25–28] and 11 case–control studies [4, 5, 8, 11, 20, 23, 24, 29–32]. Table 1 shows the baseline characteristics of the included studies. Among the eight RCTs, one study was limited by a relatively small sample size (*n* = 10), one did not describe the "Random sequence generation" and "Allocation concealment" in detail, and research assessors were not masked to group allocation. In addition, one study did not specify blinding. The other five RCTs were high-quality studies. The NOS score of 11 case–control studies was at least 7, indicating that all these studies were high-quality studies (Table 1, Fig. 2).

**Table 1:** Baseline characteristics of included studies

Study	Year	MP					PS					Design	Follow-up (years)	Outcomes*	Quality assessment
		Sample size	Age (years)	Gender (male/female)	BMI (kg/m2)	Prosthesis	Sample size	Age (years)	Gender (male/female)	BMI (kg/m2)	Prosthesis				
Lee [18]	2020	46	70 ± 7	14/32	27.4 ± 4.0	Advance	46	70 ± 7	14/32	27.4 ± 4	N/A*	RCT	1	1, 2, 3, 4, 7	Figure 2
Edelstein [19]	2020	25	67 ± 8	7/18	32.8 ± 5.8	GMK Sphere	25	64 ± 7	10/15	34.2 ± 5.8	GMK PS	RCT	2	1, 3, 4, 5, 7	Figure 2
Yuan [20]	2020	49	69.43 ± 5.97	22/27	27.81 ± 5.17	Advance	51	69.63 ± 5.72	23/28	27.59 ± 4.86	NexGen	Case–control study	5	2, 6, 9	8
Kim [21]	2009	92	69.5 ± 7.92	7/85	27.8 ± 3.15	Advance	92	69.5 ± 7.92	7/85	27.8 ± 3.15	PFC Sigma	RCT	2	1, 3, 4, 6, 8, 9	Figure 2
Hossain [22]	2011	40	72.5 ± 9.7	9/31	28.9 ± 6.2	MRK	40	68.9 ± 12.1	18/22	29.5 ± 8.1	PFC	RCT	2	1, 2, 3, 4, 5, 8, 9	Figure 2
Anderson [23]	2002	20	69 (38–89)	6/14	N/A	Advance	20	70 (47–84)	9/11	N/A	Axiom PSK	Case–control study	1	1, 9, 10	7
Bae [24]	2016	150	66.7 ± 7.1	4/120	26.4 ± 3.2	Advance	150	66.7 ± 6.5	2/136	25.9 ± 4.4	PFC Sigma	Case–control study	5	1, 2, 3, 4, 8, 9, 10, 11	7
Batra [25]	2020	53	61.7 ± 6.88	42/11	28.3 ± 3.4	Advance	53	61.7 ± 6.88	42/11	28.3 ± 3.4	Genesis II	RCT	4	1, 5, 8	Figure 2
Benjamin [26]	2018	10	64.8 (58–73)	7/3	N/A	SAIPH	10	62.4 (54 -71)	6/4	N/A	Press Fit Triathlon	RCT	1	3, 5	Figure 2
Kulshrestha [27]	2020	40	63.8 ± 6.8	11/29	27.34 ± 5.1	Advance	40	65.97 ± 6.7	17/23	26.64 ± 4.3	NexGen	RCT	2	7, 9, 11	Figure 2
Dowsey [28]	2020	29	66 ± 6.8	14/15	32.5 ± 3.6	GMK Sphere	26	65.7 ± 7.7	15/11	30.7 ± 3.8	GMK PS	RCT	1	2, 3, 4, 5, 9	Figure 2
Tan [29]	2021	12	70.8 ± 3.9	3/9	27.4 ± 2.6	Evolution	12	67.7 ± 4.9	3/9	26.9 ± 2.9	Genesis II	Case–control study	2	7	7
Lin [4]	2020	103	70.38 ± 6.37	70/33	N/A	Advance	17,893	69.32 ± 7.4274.18 ± 5.89	5/173 44/49	N/A	NexGen/NRG	Case–control study	1.6	1	8
Zhang [30]	2020	98	67.5 ± 6.5	24/74	27.3 ± 3.0	Advance	109	65.4 ± 6.2	29/80	27.6 ± 3.0	NexGen	Case–control study	1 (month)	1	8
Wang [8]	2021	126	66.92 ± 5.60	24/102	27.74 ± 4.63	Advance	126	67.15 ± 6.01	22/104	27.90 ± 4.39	NexGen	Case–control study	1	1, 2, 3, 4, 9	7
Papagiannis [32]	2016	24	70.25 ± 1.96	N/A	N/A	Advance	22	72.92 ± 1.46	N/A	N/A	RP-PS	Case–control study	2–3	1, 3, 4	7
Shi [11]	2020	290	74.5 ± 6.97	62/228	27.89 ± 3.65	Advance	237	75.4 ± 5.70	68/169	27.43 ± 3.51	NexGen	Case–control study	6–7	1, 2, 3, 4, 7, 9, 10	8
Samy [5]	2018	76	64.4 ± 10.5	29/47	29.7 (± 5.24)	Evolution	88	66.7 ± 8.61	34/54	31.3 ± 8.20	Persona	Case–control study	1	1, 7, 9, 10, 11	7
Shakespeare [31]	2006	261	76	133/128	< 30	Advance	288	78	138/150	< 30	913 PS	Case–control study	1	11	7

*Outcomes: 1. ROM, range of motion; 2. WOMAC, the Western Ontario and McMaster Universities osteoarthritis index; 3. KSS, knee society score; 4. KSFS, knee society function score; 5. OKS, Oxford knee score; 6. HSS, Hospital for Special Surgery scoring system; 7. FJS, the forgotten joint score; 8. Radiological data; 9. Complication (rate); 10. Revision rate; 11. Flexion range

*N/A, Not Applicable

### Clinical results

#### ROM and maximum flexion of the knee

**Fig. 3:**
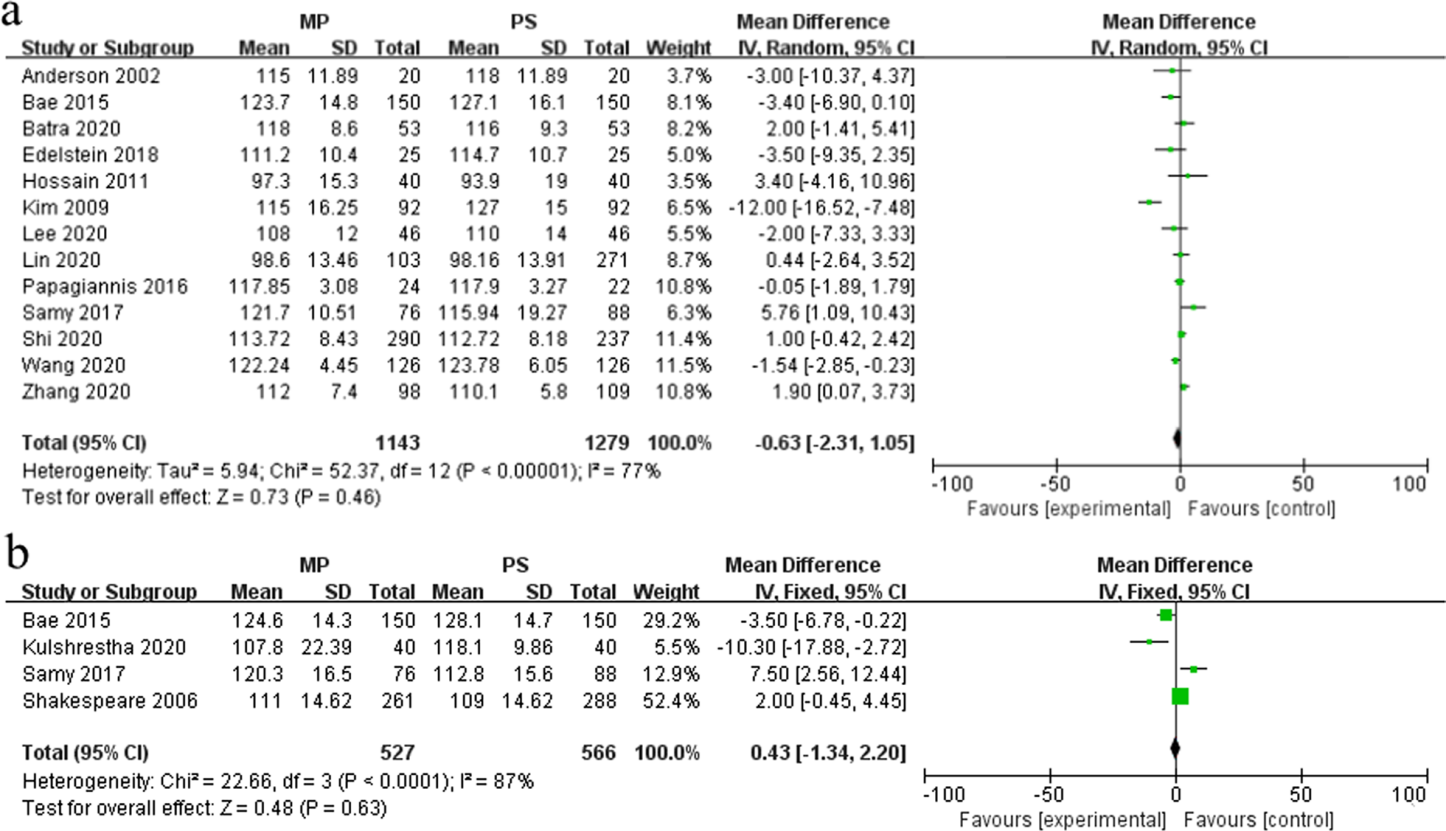
Forest plot of ROM and maximum flexion of the knee in the two groups

A total of 13 studies reported ROM [4, 5, 8, 11, 18, 19, 21–25, 30, 32]. Since high heterogeneity was indicated (*I*^2^ = 77%, *P* < 0.1), sensitivity analyses were conducted. However, sensitivity analysis showed similar results after removing the heterogeneity from the analysis. Therefore, the data were analyzed by the random-effects model. The results showed that no significant difference in ROM existed between the MP group (1143 knees) and PS group (1279 knees) (MD = − 0.63, 95% CI − 2.31–1.05, *P* = 0.46) (Fig. 3a). Four studies describing the postoperative maximum knee flexion are shown in Fig. 3b [5, 24, 27, 31]. The random-effects model was used for the meta-analysis because of significant heterogeneity (*I*^2^ = 87%, *P* < 0.1), and the results indicated that there was no significant difference in postoperative maximum flexion between the MP group (527 knees) and PS group (566 knees) (MD = 0.43, 95% CI − 1.34–2.20, *P* = 0.63).

#### Functional score

**Fig. 4:**
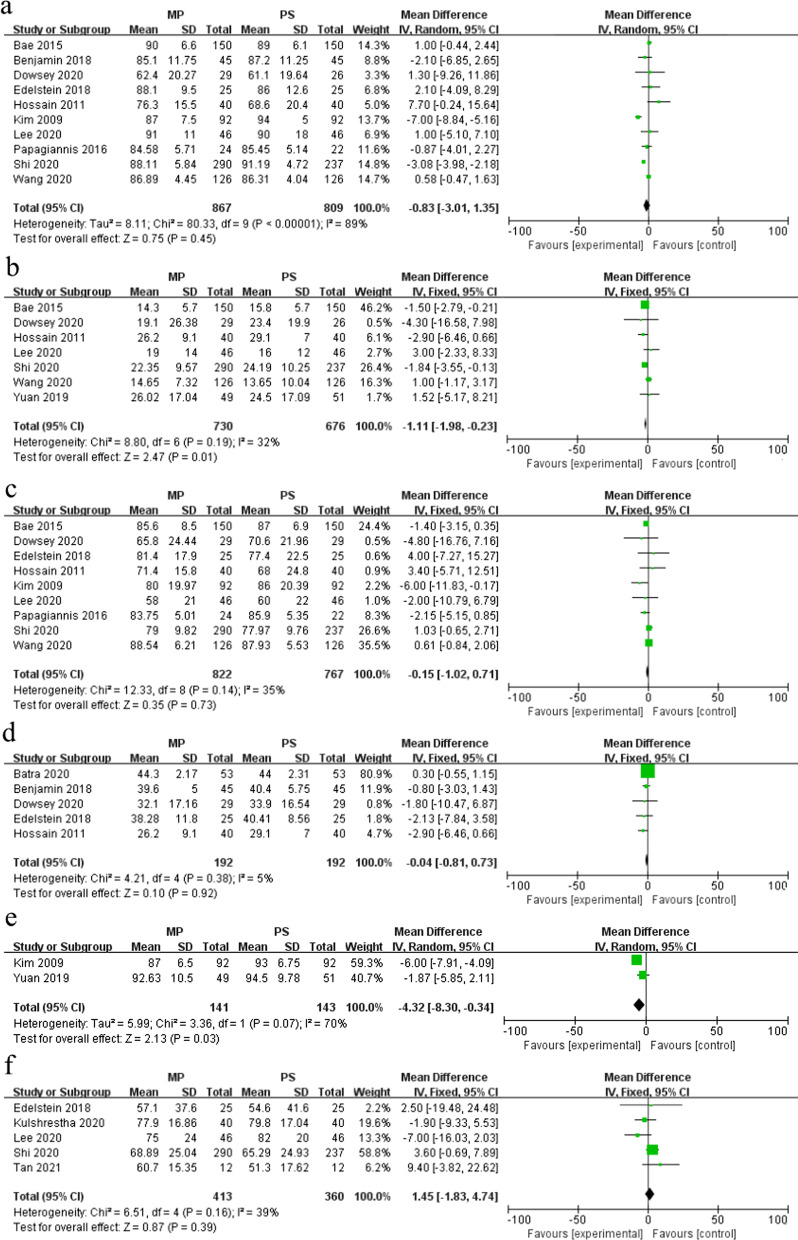
Forest plot of functional score in the two groups

The comparison of KSS between the MP group and PS group was recorded in 10 studies [8, 11, 18, 19, 21, 22, 24, 26, 28, 32].The included studies had significant heterogeneity (*I*^2^ = 89%, *P* < 0.1), and there was no change after sensitivity analysis. Random-effects model analysis showed that no significant difference in postoperative KSS existed between the MP group (867 knees) and PS group (809 knees) (MD = − 0.83, 95% CI − 3.01–1.35, *P* = 0.45) (Fig. 4a). Seven studies reported WOMAC [8, 11, 18, 20, 22, 24, 28]. The fixed-effects model (*I*^2^ = 32%, *P* = 0.19) analysis showed that the WOMAC of the MP group (730 knees) was significantly lower compared with the PS group (676 knees) (MD = − 1.11, 95% CI − 1.98 to − 0.23, *P* = 0.01) (Fig. 4b). The knee society function score (KSFS) was reported in nine studies [8, 11, 18, 19, 21, 22, 24, 28, 32]. The fixed-effects model (*I*^2^ = 35%, *P* = 0.14) analysis showed that no significant difference in postoperative KSFS existed between the MP group (822 knees) and PS group (767 knees) (MD = − 0.15, 95% CI − 1.02–0.71, *P* = 0.73) (Fig. 4c). We used the fixed-effects model (*I*^2^ = 5%, *P* = 0.38) to analyze five studies that reported OKS [17, 20, 23, 24, 26]. The results showed that the MP group (192 knees) and PS group (192 knees) had no significant difference in OKS (MD = − 0.04, 95% CI − 0.81–0.73, *P* = 0.92) (Fig. 4d). Only two studies were included in the meta-analysis regarding the HSS score [20, 21]. The random-effects model (*I*^2^ = 70%, *P* = 0.07) analysis showed that the HSS score of the MP group (141 knees) was significantly lower compared with the PS group (143 knees) (MD = − 4.32, 95% CI − 8.30 to − 0.34, *P* = 0.03) (Fig. 4e). Five studies reported the FJS [11, 18, 19, 27, 29]. The fixed-effects model (*I*^2^ = 39%, *P* = 0.16) analysis showed that there was no significant difference in FJS between the MP group (413 knees) and PS group (360 knees) (MD = 1.45, 95% CI − 1.83–4.74, *P* = 0.39) (Fig. 4f).

#### Radiographic results

**Fig. 5:**
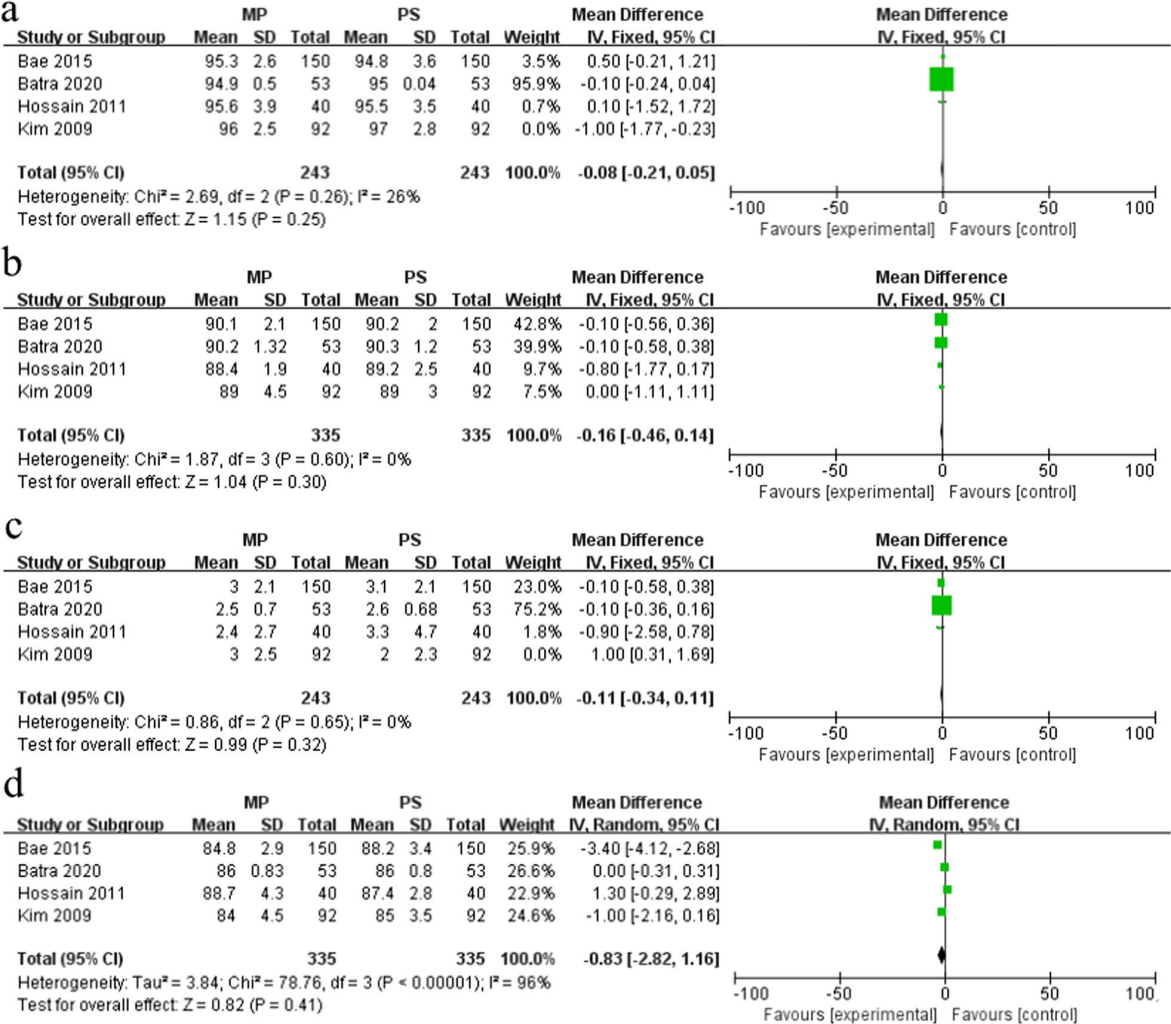
Forest plot of radiologic data in the two groups

Four studies reported radiographic results (*α*, *β*, *γ*, *δ*) [21, 22, 24, 25], while only three studies were included in the final meta-analysis of α after sensitivity analysis [22, 24, 25]. The fixed-effects model (*I*^2^ = 26%, *P* = 0.26) analysis showed that no significant difference existed between the MP group (243 knees) and PS group (243 knees) (MD = − 0.08, 95% CI − 0.21–0.05, *P* = 0.25) (Fig. 5a). Similarly, the fixed-effects model (*I*^2^ = 0%, *P* = 0.60) analysis showed that β of the MP group (335 knees) and PS group (335 knees) had no significant difference either (MD = − 0.16, 95% CI − 0.46–0.14, *P* = 0.30) (Fig. 5b). We excluded the study of Kim et al. because of high heterogeneity [21]. The fixed-effects model (*I*^2^ = 0%, *P* = 0.65) analysis of the other three studies showed that the γ of MP group (243 knees) and PS group (243 knees) was very close, and no significant difference existed between the two groups (MD = − 0.11, 95% CI − 0.34–0.11, *P* = 0.32) (Fig. 5c). Finally, the random-effects model (*I*^2^ = 96%, *P* < 0.01) analysis showed that there was still no statistical difference in δ between the two groups (MD = − 0.83, 95% CI − 2.82–1.16, *P* = 0.41) (Fig. 5d).

#### Complication rate and revision rate

**Fig. 6:**
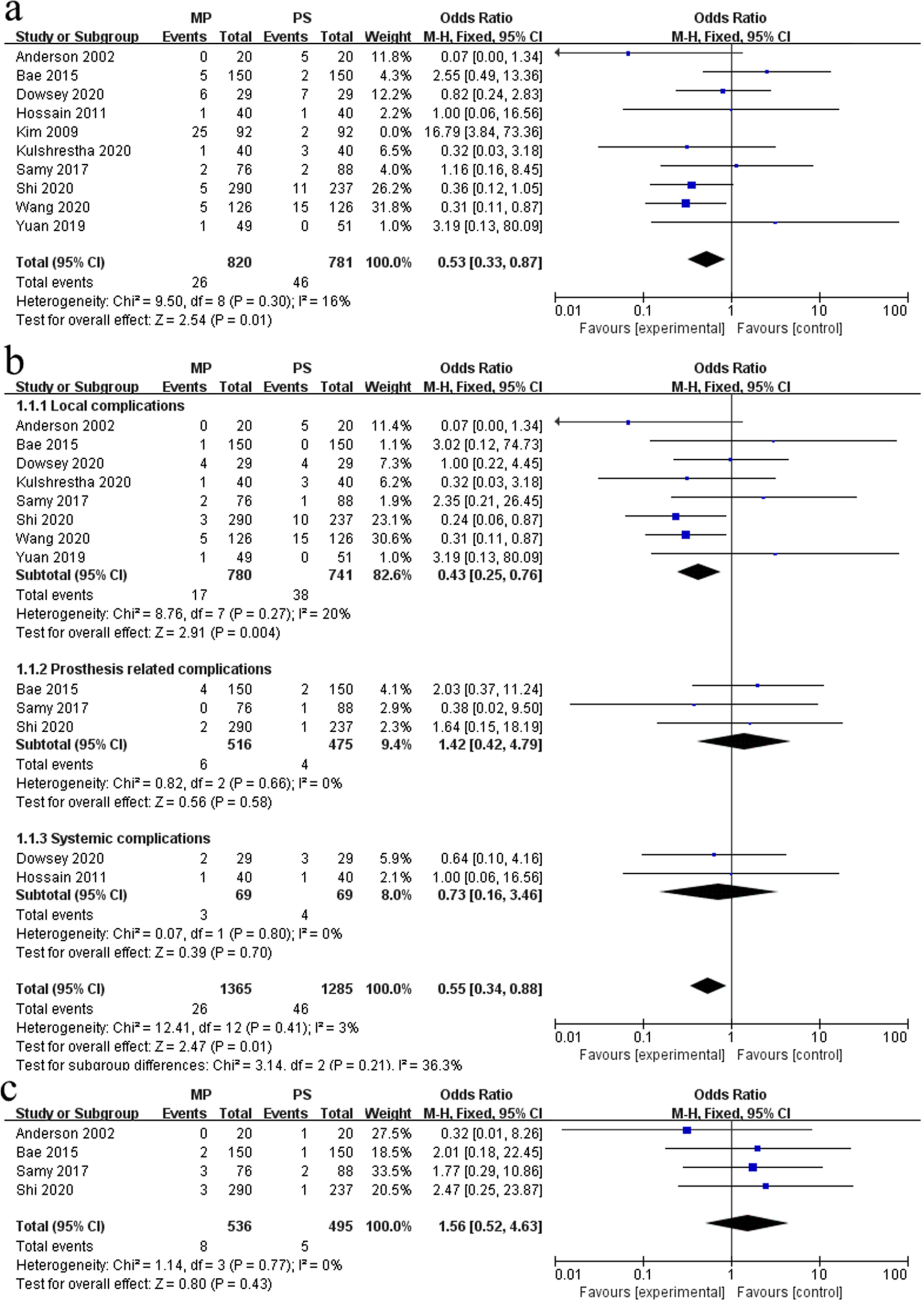
Forest plot of complication rate and revision rate in the two groups

A total of 10 studies reported postoperative complications of TKA [5, 8, 11, 20–24, 27, 28]. The study of Kim et al. [21] was excluded due to the unexplained high infection rate, and nine studies were included in the final analysis. The fixed-effects model (*I*^2^ = 3%, *P* = 0.41) analysis showed that the overall complication rate in the MP group was significantly lower compared with the PS group (OR 0.55, 95% CI 0.34–0.88, *P* = 0.01) (Fig. 6a). According to the results of subgroup analysis, a significant difference in local complication rate existed between MP-TKA and PS-TKA (OR 0.43, 95% CI 0.25–0.76, *P* = 0.004), while there was no significant difference in prosthesis-related complication rate and systemic complication rate (Fig. 6b). Four studies reported the revision rate of TKA [5, 11, 23, 24]. The fixed-effects model (*I*^2^ = 0%, *P* = 0.77) analysis showed that no significant difference in the revision rate existed between the MP group (536 knees) and PS group (495 knees) (OR 1.56, 95% CI 0.52–4.63, *P* = 0.43) (Fig. 6c).

#### Publication bias

**Fig. 7:**
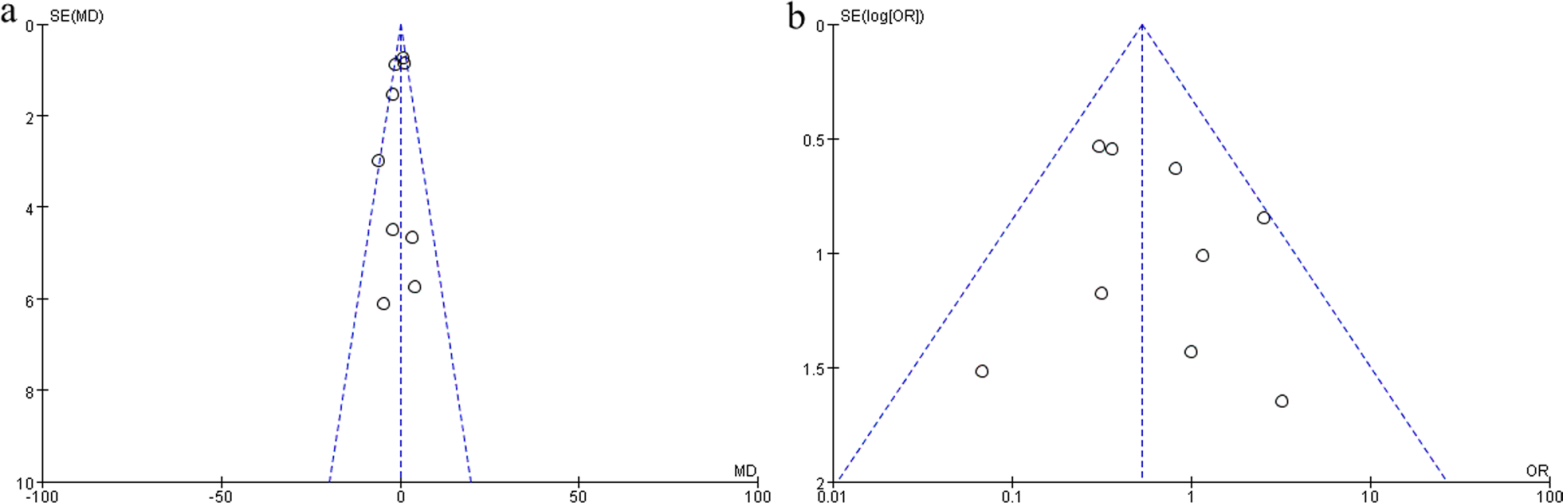
Funnel plot of KSFS (**a**) and complication rate (**b**). The location of the literature was asymmetric on both sides of the centerline, indicating that there was a publication bias in the included literature

A funnel plot was used to assess the publication bias of the KSFS (Fig. 7a) and complication rate (Fig. 7b). Visually, the distribution of all included literature on both sides of the centerline was not completely symmetrical, indicating that there might be publication bias in this analysis.

## Discussion

The results of this meta-analysis showed that the postoperative ROM was similar between the two groups. The WOMAC and HSS score after MP-TKA were significantly lower compared with PS-TKA, while there was no significant difference in KSS, KSFS, and OKS. Different from the previous three meta-analyses [12, 14, 15], we compared the position of two prosthesis. The analysis showed that the postoperative position of the prosthesis was good, and there was no significant difference. A similar meta-analysis reported that the incidence of postoperative complications of MP and PS prosthesis is similar, while this study only included four studies [13]. We added six latest studies, and the results showed that the postoperative complication rate of MP-TKA was significantly lower compared with the PS group, and there was no significant difference in revision rate between the two groups.

The PS prosthesis relies on a cam–column to achieve knee motion and stability instead of the posterior cruciate ligament, and it improves knee flexion by enhancing the femur roll-back motion [5, 6]. The MP prosthesis raises the anterior and posterior lips of the polyethylene insert to provide higher stability [22], which may limit the maximum flexion of the knee joint. Therefore, PS prosthesis has a theoretical advantage over MP prosthesis in terms of ROM and maximum knee flexion. Although several studies have reported that the ROM of the PS group is greater compared with the MP group [8, 21, 27], PS prosthesis did not show better results in comprehensive analysis, indicating that ROM was affected by many factors, and the theoretical advantage might not translate to differences in their clinical efficacy.

In this meta-analysis, the WOMAC after MP-TKA was lower than PS-TKA significantly, and there was no significant difference in KSS, OKS, and FJS, which was the same as the previous meta-analyses [13, 14]. However, it is worth considering whether the significant statistical difference of WOMAC between the two groups had clinical significance. Among the seven studies included, the maximum difference of WOMAC between the two groups was less than five points, and the comprehensive result of the meta-analysis was only 1.11 points, indicating that the WOMAC had no guiding significance in optimizing the selection of prosthesis. We thought that no significant difference in KSS and KSFS existed between the two groups because of the following three reasons. First, the postoperative effects of the two prostheses were indeed satisfactory, and there was no significant difference. Second, the scoring scale included objective indicators and subjective indicators, which were especially vulnerable to subjective disruptions. In particular, three studies reported that the same patients received different types of prostheses in bilateral TKA [18, 21, 25]. Therefore, it was difficult for patients to clearly distinguish the function and feel of each knee joint. Third, the scoring systems, such as KSS, were affected by the upper limit effect, resulting in the reduced sensitivity of the system, and it could not accurately reflect the differences between groups [33]. Bae et al. [24] believed that the maximum flexion is the main determinant of the FJS, while Peng et al. [34] thought that pain is an important factor dramatically impairing the quality of life in Chinese KOA patients, and that relief of pain is the reason for the high FJS rather than the recovery of function. At present, only a few studies adopt FJS, so the main influencing factors of FJS are unclear. Therefore, although FJS has a high sensitivity, the meta-analysis showed that no major differences were found among the groups the two groups.

Thompson et al. [35] found that when the knee joint is straightened, variability in rotational alignment of femoral and tibial components will lead to improper joint kinematics, and poor alignment will also change the patella trajectory, resulting in AKP [36]. Therefore, we decided to determine the accuracy of the prosthesis position by *α*, *β*, *γ*, and *δ*. Only in the study of Bae et al. [24], *δ* in the MP group was significantly smaller compared with the PS group, while it did not affect the postoperative results. Apart from it, other angles were close to a normal angle [21, 22, 25]. Meta-analysis showed that *α*, *β*, *γ*, and *δ* between the two groups were not significantly different in the four studies. Thus, we believed that the design of the prosthesis would not have a significant impact on the position of the prosthesis.

Different from previous meta-analyses [12, 14, 15],we paid more attention to the postoperative complication rate and revision rate of MP-TKA and PS-TKA. The study of Kim [21]et al. was terminated early because of the high infection rate (6%). At the end of the study, 25 complications (27.2%) occurred in the MP group, including infection (6%), recurrent effusion (5%), flexion contracture (2%), supracondylar fracture (0.5%), and skin edge necrosis (0.5%), while only two complications occurred in the PS group. Although the authors explained that the high postoperative complication rate had nothing to do with the “surgeon-specific factor,” the failure in the restoration of normal kinematics with the MP prosthesis might lead to recurrent effusion and infection; hence, we remained skeptical about the conclusions of the study. Therefore, it was excluded from the analysis.

The final meta-analysis showed that the overall complication rate of MP-TKA was significantly lower compared with PS-TKA. Further subgroup analysis found that the difference was mainly concentrated in the local complications of the knee joint, and no significant difference in the frequency of prosthesis-related complications and systemic complications was observed between the two groups. The complications in the PS group were mainly PCC (43.5%) and AKP/pain (21.7%), while those in the MP group were only 15.4% and 7.7%, respectively, which confirmed the concern that prosthesis design defects might bring high complication rate. Prosthetic design features are an important cause of patellofemoral joint problems [37], and it is difficult to make up for the inherent flaw of prosthesis by improving surgical techniques. In the study of Wang et al., there were only two cases of PCC in the MP group and nine cases in the PS group, which was much higher compared with the MP group (7.1% vs. 1.6%). MP prosthesis could maximize the recovery of the natural movement of the knee, which was conducive to reducing patellofemoral joint pressure. In addition, the MP prosthesis has a longer femoral trochlear groove and smoother patellar trajectory, which could explain why the patellofemoral joint complications of MP prosthesis were significantly lower compared with the PS prosthesis.

The incidence of complications related to MP-TKA and PS-TKA prostheses was 1.2% and 0.8%, respectively, and there was no significant difference (*P* = 0.58). The main complications were aseptic loosening and periprosthetic joint infection (PJI). These incidents could be reduced by improving the surgical technique and postoperative nursing quality. The incidence of systemic complications, such as pulmonary embolism and acute myocardial infarction, was lower, and there was no significant difference, indicating that the two types of prostheses were safe. In conclusion, MP prosthesis is significantly better compared with PS prosthesis in terms of local complications.

In this meta-analysis, there was no significant difference in revision rate between the MP-TKA and PS-TKA groups (1.5% vs. 1.0%, *P* = 0.28). Among the four studies included in the analysis, the main causes of MP prosthesis revision were infection (0.7%), including one case of deep infection and three cases of PJI, knee instability (0.4%), aseptic loosening of femoral components (0.2%), and pain (0.2%), while those reasons for PS prosthesis were mainly infection (0.4%), knee instability (0.2%), aseptic loosening of tibial components (0.2%), and PCC (0.2%). We held the opinion that except for one case of revision due to PCC, the need for revision was not correlated with the prosthesis design, but rather with surgical technology or postoperative care. Therefore, no significant difference was found in the revision rate between the two groups.

There are several limitations of this meta-analysis. Firstly, among the 19 included studies, 11 were retrospective studies and eight were RCTs, indicating that inherent bias was inevitable and the level of evidence provided was limited. Secondly, the main outcomes were patient-reported outcome measures (PROMs); proprioception and gait analysis were rarely studied at present because of high technical requirements. Thirdly, the longest follow-up time included in the meta-analysis was 7 years, and the data, such as revision rate, need to be further analyzed by long-term follow-up data. Fourthly, in different studies, the heterogeneity caused by perioperative treatment of patients, surgical techniques of operators, and even rehabilitation guidance of nurses could not be controlled.

## Conclusions

This meta-analysis comprehensively compared the postoperative efficacy and safety of MP-TKA and PS-TKA. Although there was no significant difference in ROM, KSS, OKS, FJS, radiographic results, and revision rate between the MP-TKA and PS-TKA, lower WOMAC, HSS, and complication rate were observed in the MP-TKA group. Taking these results together, we conclude that MP prosthesis has a better clinical effect and significantly lowered complication rate than PS prosthesis.

## Data Availability

Not applicable.
